# Verifications

**Published:** 1889-05

**Authors:** Oran W. Smith

**Affiliations:** Union Springs, N. Y.


### VERIFICATIONS.

EDITORS HOMŒOPATHIC PHYSICIAN:—Perhaps the few items I send you may aid you in your commendable work. If every homœopathic physician would contribute of his reliable knowledge and experience, what a valuable store-house of facts your Repertory would be—not that I am depreciating its value at all, but there is so much that is *garnered up* in the experience of most active and observing M. D's.

The "verifications" that I send are of symptoms less frequently noticed, and therefore worthy of record. Those symptoms that are the results of poisoning or large doses or experimental doses or continued or excessive use, are those that occur under Æsculus hip., Pastinaca sat. (wild parsnip), Acetic acid (hard cider), Atropia sulph., Tabacum, Ruta, Vespa, Ars. iod., and were due, without doubt, to the drugs and substances named. "C. S." following the name of a remedy indicated *cured symptoms.* These symptoms were prominent in the cases cured, and the cures were unquestionably due to the medicine. They are mentioned only where the single remedy was used and the result so prompt as to eliminate doubt.

### PATHOGENETIC.

ACETIC ACID (hard cider, excessive use): Dark, bluish-red tumor as large as a good-sized chestnut on left verge of anus.

ÆSCULUS HIP. (child three years old ate several): Vomiting of a watery, colorless fluid. Posterior half of tongue covered with a thick, yellow fur. Anterior half coated thinly white, and studded with minute red points.

PASTINACA SAT. (wild parsnip; child two years old poisoned by handling and eating): Vomiting of milk in large, hard curds. Externally skin was red, hot, swollen. Scrotum, eyelids œdematous and translucent to lamp-light, seemingly distended with fluid. Blebs and blisters on hands and fingers.

TABACUM (excessive smoking): Prolapsus ani. Great drowsiness during day when trying to read or study.

VESPA (stung three times about head and neck): Trembling of hands whenever trying to use them. Dilated pupils. Vision indistinct. Head seemed to expand whenever moving it. This sensation of expansion began in nape. Sensation of constriction as though skull were too small for brain.

ATROPIA SULPH. (1-40 grain, experimentally): Great dryness of mouth, tongue and fauces, yet no thirst. Strong desire to urinate, yet almost complete loss of expulsive power. In dreams, great contempt for religious and sacred matters. Headache worse when lying down. Vision for smaller letters impaired. Larger letters appeared as if printed with yellow ink, with a narrow border of black. While writing, pen seemed to have a double point.

RUTA GRAV. (after long-continued use internally of 3d dec.): Ganglionic swelling on front of left wrist.

VERIFIED.

CINNABAR: Headache, frontal, commencing in the morning soon after getting out of bed, relieved by pressure of the hands upon the forehead.

BISMUTH SUB. NIT.: Pain in stomach relieved by bending backward.

NAT. MUR.: Painful eruption in border of hair on right temple.

NUX VOM.: Coryza, from left nostril. Fluent during day, dry at night.

KOBALT. PULS. Sep. ZINC: Pain in back relieved by walking.

ELAPS. COR.: Inflammation, soreness, and intense itching of left eye.

NAT. SULPH.: Pain in right hypochondrium. Worse lying on left side. When lying on left side, a dragging sensation in right hypochondrium.

SPIGELIA: Pains as if needles were thrust into right eyeball.

LACH.: During chill wants to be held close.

CALC. CARB.: Cough excited by least current of air, even by a person passing near.

CARB. VEG.: Soon after eating, belching followed by burning in the stomach.

THUJA: Cough during day. None after lying down. Pain on inner side of left arm from elbow to hand. Worse forenoon. Begins three A. M.

CACT. GR.: Sensation of a band of three fingers' width constricting the epigastric region; felt especially before stool.

BORAX: Apthæ inside of lower lip, on tip of tongue. Awakens very early in the morning. Hunger.

SANG.: Pain shooting from lower part of left chest to left shoulder.

AGAR. MUSC.: Yellow spots before vision when looking at anything white.

CLEMATIS: C. S. Crawling, creeping sensations in scrotum.

EUPHRASIA: C. S. Cough loose through day, dry at night.

SANG.: C. S. Headache concentrating in a small spot over right eye. The eye itself becomes red and sore, yet hard pressure upon eye relieves.

ARNICA: C. S. Frontal headache, aggravated by jar, noise, and moving. Pain extends to eyeballs. Pillow feels hard as a stone.

LAUROCERASUS: C. S. Stitching pain from left scapula through left side to infra-mammary region. Ache in forehead, with cold sensation as if cold wind were blowing upon it.

CARB. VEG.: C. S. Excessive hunger at night. Must eat to appease it. Desire to urinate whenever arising from sitting posture. Cheerful forenoons, despondent evenings.

CARBOLIC ACID: C. S. Excessive accumulation of gas in stomach. Belching.

BISMUTH SUB. NIT.: C. S. Eructations tasting of food eaten twenty-four hours before.

SANG: C. S. Red streak through centre of tongue.

MAG. PHOS.: C. S. Toothache, second molar, lower jaw, left side. Steady pain with shootings.

IRIS. VERS.: C. S. Purging and vomiting at the same time.

NAT. SULP: C. S. When lying on left side pulling sensation in right hypochondrium.

CALC. PHOS: C. S. Pains in the scars of old abscesses.

ACTEA. RAC: C. S. Sensation as if her head was full of little beings that kept at work. Sensation as if she were in a cloud (mentally). Beating and fullness in sides of neck. Feels as if blood all left the heart and went to head.

ARG. MET.: C. S. Left inguinal region occupied with a hard red swelling. Very painful. Pain follows Poupart's ligament over top of hip bone to back and kidney. Marked sensation of tension and drawing in lifting region.

CINA.: C. S. Involuntary urination when under excitement or emotion. Wetting bed at night (child).

PHYTOLACCA: C. S. Hunger at the onset of a chill. During chill soles of feet become very cold. Sharp shooting pains in a broken leg. Pains begin in heel and shoot upward to hip. Leg jerks upward.

BAPTISIA: C. S. Numbness and tingling in the whole of left side. A distinct sensation of crepitation in left wrist when bending the hand. Shooting pains about the heart. Great anxiety and fear of an incurable heart disease. Tongue coated upon right side.

MERC-IOD-FLAV: C. S. Vertigo, with sensation as if he was walking upon the air.

SABINA: C. S. Uterine hemorrhage with pain in the back.

LACHESIS: C. S. Sickening pain in left hypochondrium going through to back, with a sensation as if a cord were drawn tight about the left side.

STAPH.: C. S. Sensation of stiffness and contraction in hollows of knees. Dribbling of urine.

CALC-CARB.: C. S. Cough with bursting pain in occiput; with loss of taste and smell; redness of tip of nose. Cough with soreness through lower part of abdomen; holds abdomen with hands when coughing. With cough, cutting pain in right side of throat, which aches afterward. Severe aching in left mastoid process, with severe shooting pains extending upward and downward when moving the head.

NAT-MUR.: C. S. Chill beginning in elbows and knees.

THUJA: C. S. Cutting, shooting, and squeezing pains in region of left ovary.

NUX-VOM.: C. S. Sensation under middle of sternum, like a lump of hot lead as large as two fists.

ORAN W. SMITH.

UNION SPRINGS, N. Y.

